# Influence of Metabolic Risk Factors on the Risk of Bacterial Infections in Hepatitis B-Related Cirrhosis: A 10-Year Cohort Study

**DOI:** 10.3389/fmed.2022.847091

**Published:** 2022-04-14

**Authors:** Qiao Yang, Yifan Tong, Borui Pi, Hong Yu, Fangfang Lv

**Affiliations:** ^1^Department of Infectious Diseases, Sir Run Run Shaw Hospital, School of Medicine, Zhejiang University, Hangzhou, China; ^2^Department of General Surgery, Sir Run Run Shaw Hospital, School of Medicine, Zhejiang University, Hangzhou, China

**Keywords:** bacterial infections, body mass index, diabetes, liver cirrhosis, high-density lipoproteins, triglycerides

## Abstract

**Aim:**

The effect of metabolic factors on the risk of bacterial infections (BIs) in patients with hepatitis B virus (HBV)-related cirrhosis has not been demonstrated. This study aimed to explore specific metabolic factors associated with the BIs in these patients.

**Methods:**

A population-based cohort of 471 patients with HBV-related cirrhosis was retrospectively enrolled between 2009 and 2019. The primary end point was the incidence of BIs during hospitalization, which were compared according to the metabolism-related indicators, namely, presence of diabetes, level of high-density lipoprotein cholesterol (HDLC) and triglyceride, and body mass index (BMI). The propensity score matching (PSM) was adopted to eliminate baseline discrepancies.

**Results:**

Compared with the non-diabetic group, the incidences of BIs were higher in the diabetic group before and after PSM (*p* = 0.029 and *p* = 0.027). Similar results were found in the low HDLC group as compared with the normal HDLC group before and after PSM (*p* < 0.001 and *p* = 0.025). Further analysis showed that the incidences of BIs in patients with low HDLC alone were lower than patients with both low HDLC and diabetes before and after PSM (*p* = 0.003 and *p* = 0.022). Similarly, the incidence of BIs in patients with diabetes alone was lower than those in patients with both low HDLC and diabetes both before and after PSM (*p* = 0.002 and *p* = 0.018). However, neither triglyceride nor BMI level was related to BIs in our cohort.

**Conclusion:**

In patients with HBV-related cirrhosis, the presence of diabetes and low level of HDLC were risk factors of BIs, showing a synergistic effect.

## Introduction

Bacterial infection (BI) is a frequent and challenging complication in patients with cirrhosis worldwide (1). Given the asymptomatic onset and uncertain infected lesion, early diagnosis of BIs in patients with cirrhosis requires an in-depth understanding of the clinical condition and detailed physical and imaging examinations. In diseased livers such as cirrhosis, episodes of BIs sometimes contribute to acute hepatic decompensation including acute-on-chronic liver failure (ACLF) *via* multifactorial mechanisms (2). Therefore, the management of patients with cirrhosis requires careful attention to BIs (3).

The liver is the central organ of metabolism, participating in a variety of biological processes including glucose and lipid metabolisms, coagulation function regulation, and detoxification (4). The prevalence of metabolic syndrome, comprising type 2 diabetes, hypertension, obesity, and dyslipidemia, has been increasing in recent years, which has become a public health problem. Abnormalities in glucose metabolism are common in patients with cirrhosis, with type 2 diabetes mellitus (DM) affecting approximately one-third of the patients (5, 6). Specifically, diabetes is associated with the occurrence of major complications of cirrhosis, including BIs, as well as mortality (7). Several clinical studies showed that the synergy of diabetes and cirrhosis predisposed patients to BIs (8). Therefore, screening for diabetes should be initiated during hospitalization in patients with cirrhosis.

Alterations of liver function are associated with modifications of circulating lipids. Progressive dyslipidemia occurs in liver cirrhosis and is related to increased mortality. The prevalence of the fatty liver disease is approximately one-quarter of the general population (9, 10). Where HBV infection is endemic, the concomitant presence of fatty liver disease in patients with chronic hepatitis B is common, ranging from 14 to 70% (10, 11). A previous study also demonstrated that steatohepatitis is an independent predictor of significant fibrosis and advanced fibrosis (10). In addition to hepatic steatosis, the evidence for dyslipidemia as a risk factor for the development of HBV-related fibrosis progression has been well described (12). A recent study further showed that cirrhotic patients with a lower level of high-density lipoprotein cholesterol (HDLC) were prone to BIs and death since the immune modulatory function of HDLC on monocytes was impaired in this setting (13). In brief, studies in HBV-infected populations have shown that hepatic steatosis, DM, obesity, dyslipidemia, or a combination of metabolic risk factors are associated with the risk of cirrhosis. However, the effect of metabolic factors on BIs has not been fully understood in patients with HBV-related cirrhosis. Therefore, the aim of this study was to explore potential factors associated with BIs in patients with cirrhosis, which might facilitate physicians’ decision-making clinically.

## Materials and Methods

### Patient Selection

**FIGURE 1:**
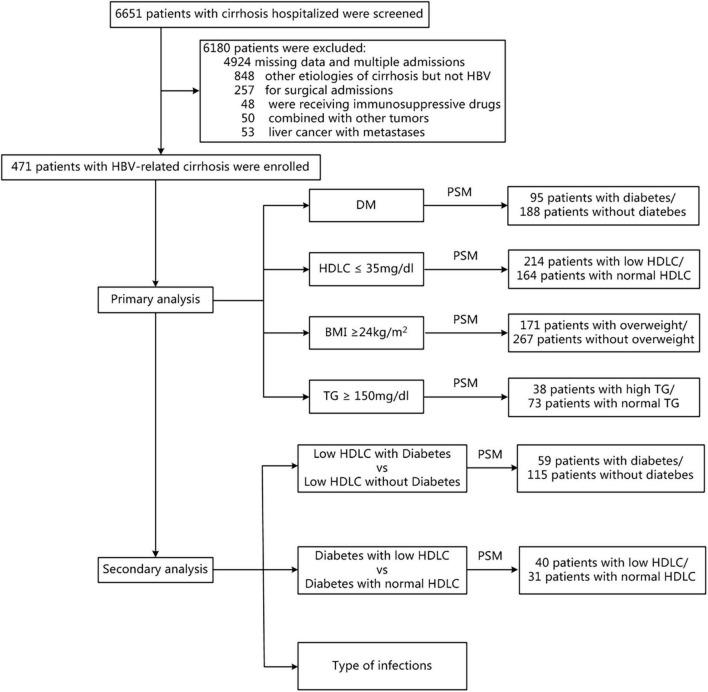
Flow chart of patient selection. BMI, body mass index; DM, diabetes mellitus; HDLC, high-density lipoprotein cholesterol; PSM, propensity score matching; TG, triglyceride.

This study was conducted in accordance with the principles of the Declaration of Helsinki and was approved by the Ethics Review Committee of Sir Run Run Shaw Hospital, Zhejiang University, China. We retrospectively enrolled a population-based cohort of 471 patients with HBV-related cirrhosis in the Sir Run Run Shaw hospital between 2009 and 2019. Retrospectively, demographic and clinical data of patients meeting the inclusion criteria were collected from our database (Figure 1). The diagnosis of HBV-related cirrhosis was made according to liver biopsy, endoscopic signs of portal hypertension, radiological evidence of liver nodularity, or clinical evidence of prior hepatic decompensation in patients with chronic HBV-infected liver diseases (3). Patients with hepatitis B-related hepatocellular carcinoma with Barcelona Clinic Liver Cancer Stage A who were stable after surgery or percutaneous ablation were also included in this study. Patients were excluded from this study if any of the following criteria were met: (1) cirrhosis caused by other reasons (e.g., hepatitis C virus infection, alcohol use disorder, autoimmune liver diseases, and schistosomiasis); (2) active solid organ malignancy; (3) postsplenectomy, receiving glucocorticoid or immunosuppressive medications; (4) receiving operation during this hospitalization; and (5) crucial data missing.

### Data Collection

Demographic characteristics, medical history, laboratory outcomes, metabolism-related indicators, and model for end-stage liver disease (MELD) scores were recorded. The primary end point was the incidence of BIs during hospitalization, which was diagnosed according to the conventional criteria (Supplementary Table 1) (14). Initially, all subsets’ regression was performed to identify the metabolism-related risk factors in terms of BIs. Then, the incidences of BIs were compared in different groups according to the following metabolic risk factors: (a) obesity, defined as body mass index (BMI) ≥ 24 kg/m^2^ for Asian patients; (b) hypertriglyceridemia, defined as a serum triglyceride (TG) level ≥ 150 mg/dl; (c) DM, defined as a fasting glucose level of ≥ 126 mg/dl or prior diagnosis of DM and/or treatment with antidiabetic agent or insulin; (d) decrease of HDL level, defined as a serum HDL level ≤ 35 mg/dl. Extrahepatic diseases included cardiovascular, respiratory, and/or kidney diseases. All the enrolled patients were reviewed by two investigators, and any discrepancy between the two investigators was referred to a senior liver disease specialist for adjudication. The PSM with a ratio of 1:2 was adopted to eliminate baseline discrepancies and avoid losing too many samples. Eventually, the types of BIs were also compared.

### Statistical Analysis

The continuous variables were expressed as medians with ranges, while categorical data were presented as numbers with proportions. Correspondingly, the Mann-Whitney *U*-test was used to compare continuous variables, while the Pearson’s chi-squared test or Fisher’s test was used to compare categorical data as appropriate. The *p*-value less than 0.05 in two-tailed analyses was defined as statistical significance. All statistical analyses were performed using R software (Version 3.4.1).^1^

## Results

**FIGURE 2:**
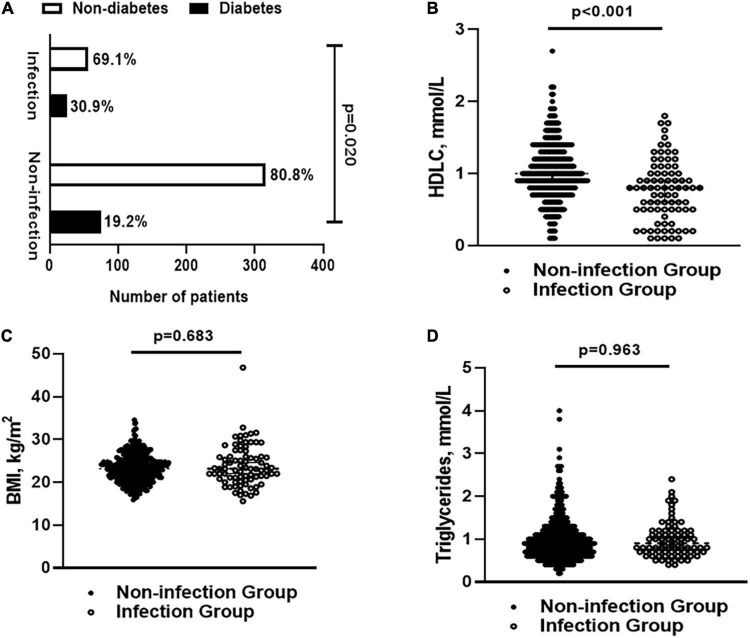
**(A)** The percentage of patients with diabetes and non-diabetes in infection and non-infection groups; **(B)** levels of HLDC in infection and non-infection groups; **(C)** BMI in infection and non-infection groups; and **(D)** levels of triglyceride in infection and non-infection groups. BMI, body mass index; HDLC, high-density lipoprotein cholesterol.

Totally, 471 patients were enrolled in this study. The demographic characteristics, metabolism-related indicators, laboratory results, and features of BIs were summarized in Supplementary Table 2. We performed full subset regression to assess the significance of several metabolic factors (e.g., BMI, diabetes, HDLC, and TG) for the incidence of BIs. The results showed that diabetes and HDLC seemed to be more relevant to the incidence of BIs. Further analysis confirmed that patients with diabetes and low HDLC had an increased incidence rate of BIs (*p* = 0.020 and *p* < 0.001, Figures 2A,B). Besides, there was no significant difference in BMI/TG in patients with or without hospital infections (*p* = 0.683 and *p* = 0.963, Figures 2C,D).

In total, 100 of 471 patients had coexistence of diabetes. By PSM, 188 patients in the non-diabetic group and 95 patients in the diabetic groups were matched (Table 1). Compared with the non-diabetic group (15.1% and 13.8%), the incidences of BIs were significantly higher in the diabetic group (25.0% and 25.3%) before and after PSM (*p* = 0.029 and *p* = 0.027). With regard to HDLC, the incidences of BIs were 22.4% in the low HDLC group (*n* = 277) and 9.8% in the normal HDLC group (*n* = 194), respectively (*p* < 0.001). Adjusted by age, sex, presence of hepatocellular carcinoma, level of triglycerides, laboratory investigations, and MELD score, the incidence of BIs remained to be higher in the low HDLC group (17.8%) than that in the normal HDLC group (9.1%) (*p* = 0.025) (Table 2). As expected, the incidence of infection was comparable between groups stratified by BMI and TG (Supplementary Tables 3, 4).

**TABLE 1:** Clinical characteristics stratified by DM.

	Before propensity score matching			After propensity score matching		
	Non-diabetes group (*n* = 371)	Diabetes group (*n* = 100)	*p*-value	Non-diabetes group (*n* = 188)	Diabetes group (*n* = 95)	*p*-value
Sex			0.562			0.922
Male	280 (75.5)	72 (72.0)		138 (73.4)	71 (74.7)	
Female	91 (24.5)	28 (28.0)		50 (26.6)	24 (25.3)	
Age, years			0.114			0.976
≥60	129 (34.8)	44 (44.0)		81 (43.1)	40 (42.1)	
<60	242 (65.2)	56 (56.0)		107 (56.9)	45 (57.9)	
BMI, kg/m^2^			0.315			0.907
≥24.0	144 (38.8)	45 (45.0)		84 (43.1)	41 (43.2)	
<24.0	227 (61.2)	55 (55.0)		104 (56.9)	54 (56.8)	
Extra-hepatic diseases			0.061			0.715
Present	86 (23.2)	33 (33.0)		52 (27.7)	29 (30.5)	
Absent	285 (76.8)	67 (67.0)		136 (72.3)	66 (69.5)	
Hepatocellular carcinoma			0.578			1.000
Present	151 (40.7)	37 (37.0)		78 (41.5)	39 (41.1)	
Absent	220 (59.3)	63 (63.0)		111 (58.5)	56 (58.9)	
HDLC, mmol/L			1.000			1.000
Normal	153 (40.7)	41 (41.0)		78 (41.5)	39 (41.1)	
Low	218 (59.3)	59 (59.0)		110 (58.5)	56 (58.9)	
Triglycerides, mmol/L			0.935			1.000
High	33 (8.9)	8 (8.0)		16 (8.5)	8 (8.4)	
Normal	338 (91.1)	92 (92.0)		172 (91.5)	87 (92.6)	
ALT, IU/L			0.910			0.979
≥2-fold normal upper limit	61 (16.4)	16 (16.0)		31 (16.5)	15 (15.8)	
0–2-fold normal upper limit	77 (20.8)	19 (19.0)		36 (19.1)	19 (20.0)	
Normal	233 (62.8)	65 (65.0)		121 (64.4)	61 (64.2)	
AST, IU/L			0.191			0.692
≥2-fold normal upper limit	68 (18.3)	20 (20.0)		32 (17.0)	19 (20.0)	
0–2-fold normal upper limit	112 (30.2)	21 (21.0)		47 (25.0)	20 (21.1)	
Normal	191 (51.5)	59 (59.0)		109 (58.0)	56 (58.9)	
MELD score			0.303			0.946
Median	8.0	8.0		8.0	8.0	
Interquartile range	5–12	5–12		5–11	5–12	
Infection			0.029			0.027
Present	56 (15.1)	25 (25.0)		26 (13.8)	24 (25.3)	
Absent	315 (84.9)	75 (65.0)		162 (86.2)	71 (74.7)	

*Data are presented as the number with percentage or median with interquartile range. DM, diabetes mellitus; ALT, alanine transaminase; AST, aspartate aminotransferase; BMI, body mass index; HDLC, high-density lipoprotein cholesterol; MELD, model for end-stage liver disease.*

**TABLE 2:** Clinical characteristics stratified by HDLC.

	Before propensity score matching			After propensity score matching		
	Low HDLC group (*n* = 277)	Normal HDLC group (*n* = 194)	*p*-value	Low HDLC group (*n* = 214)	Normal HDLC group (*n* = 164)	*p*-value
Sex			0.001			0.135
Male	191 (69.0)	161 (83.0)		157 (73.4)	132 (80.5)	
Female	86 (31.0)	33 (17.0)		57 (26.6)	33 (19.5)	
Age, years			0.017			0.175
≥60	89 (32.1)	84 (43.3)		73 (34.1)	68 (41.5)	
<60	188 (67.9)	110 (56.7)		141 (65.9)	96 (58.5)	
BMI, kg/m^2^			0.006			0.129
≥24.0	126 (45.5)	63 (32.5)		92 (43.0)	57 (34.8)	
<24.0	111 (54.5)	131 (67.5)		122 (57.0)	107 (65.2)	
Diabetes			1.000			0.601
Present	59 (21.3)	41 (21.1)		49 (22.9)	33 (20.1)	
Absent	218 (78.7)	153 (78.9)		165 (77.1)	131 (79.9)	
Extra-hepatic disease			0.449			1.000
Present	74 (26.7)	45 (23.2)		52 (24.3)	40 (24.4)	
Absent	203 (73.3)	149 (76.8)		166 (75.7)	124 (75.6)	
Hepatocellular carcinoma			<0.001			0.085
Present	91 (32.9)	97 (50.0)		83 (38.8)	79 (48.2)	
Absent	186 (67.1)	97 (50.0)		131 (71.2)	85 (51.8)	
Triglycerides, mmol/L			0.034			0.372
High	31 (11.2)	10 (5.2)		18 (8.4)	9 (5.5)	
Normal	246 (88.8)	184 (94.8)		196 (91.6)	155 (94.5)	
ALT, IU/L			<0.001			0.372
≥2-fold normal upper limit	63 (22.7)	14 (7.2)		25 (11.7)	13 (7.9)	
0–2-fold normal upper limit	52 (18.8)	44 (22.7)		46 (21.5)	42 (25.6)	
Normal	162 (58.5)	136 (70.1)		143 (66.8)	109 (66.5)	
AST, IU/L			<0.001			0.174
≥2-fold normal upper limit	75 (27.1)	13 (6.7)		30 (11.7)	13 (7.9)	
0–2-fold normal upper limit	71 (25.6)	62 (32.0)		63 (29.4)	42 (32.9)	
Normal	131 (47.3)	119 (61.3)		121 (56.5)	109 (59.1)	
MELD score			<0.001			0.068
Median	9.0	7.0		9.0	7.0	
Interquartile range	6–13	5–10		5–12	5–11	
Infection			<0.001			0.025
Present	62 (22.4)	19 (9.8)		38 (17.8)	15 (9.1)	
Absent	215 (77.6)	175 (90.2)		176 (82.2)	149 (90.9)	

*Data are presented as the number with percentage or median with interquartile range. ALT, alanine transaminase; AST, aspartate aminotransferase; BMI, body mass index; HDLC, high-density lipoprotein cholesterol; MELD, model for end-stage liver disease.*

Given that the aforementioned results suggested that both diabetes and low level of HDLC were associated with increased risk of BIs, further investigations were performed to explore whether the synergistic effect existed. In patients with a low level of HDLC (*n* = 277), there were 218 patients without diabetes and 59 patients with diabetes, respectively (Table 3). The incidences of BIs in patients with low HDLC alone were significantly lower than patients with low HDLC and diabetes both before and after PSM (*p* = 0.003 and *p* = 0.022). Likewise, the incidence of BIs (7.3%) in patients with diabetes alone (*n* = 41) was dramatically lower than 18.3% in patients with low HDLC and diabetes (*n* = 59) (*p* = 0.002) (Table 4). To exclude the confounding effects, the BMI, laboratory investigations, and MELD score showed no difference between the two groups after PSM. In this setting, the incidences of BIs were 32.5% in the low HDLC (*n* = 40) and diabetic group and 6.5% in the diabetic group alone (*n* = 31), respectively (*p* = 0.018).

**TABLE 3:** Clinical characteristics stratified by DM in patients with low HDLC.

	Before propensity score matching			After propensity score matching		
	Non-diabetes group (*n* = 218)	Diabetes group (*n* = 59)	*p*-value	Non-diabetes group (*n* = 115)	Diabetes group (*n* = 59)	*p*-value
Sex			1.000			0.753
Male	150 (68.8)	41 (69.5)		84 (73.0)	41 (69.5)	
Female	68 (31.2)	18 (30.5)		31 (27.0)	18 (30.5)	
Age, years			0.628			1.000
≥60	68 (31.2)	21 (35.6)		40 (34.8)	21 (35.6)	
<60	150 (68.8)	38 (64.4)		75 (65.2)	38 (64.4)	
BMI, kg/m^2^			0.169			1.000
≥24.0	94 (43.1)	32 (54.2)		63 (54.8)	32 (54.2)	
<24.0	124 (56.9)	27 (45.8)		48 (45.2)	27 (45.8)	
Extra-hepatic disease			0.364			0.776
Present	55 (25.2)	19 (32.2)		41 (35.7)	19 (32.2)	
Absent	163 (74.8)	40 (67.8)		74 (64.3)	40 (67.8)	
Hepatocellular carcinoma			0.556			1.000
Present	74 (33.9)	17 (28.8)		34 (29.6)	17 (28.8)	
Absent	144 (66.1)	42 (71.2)		81 (70.4)	42 (71.2)	
Triglycerides, mmol/L			0.962			1.000
High	25 (11.5)	6 (10.2)		11 (9.6)	6 (10.2)	
Normal	193 (88.5)	53 (89.8)		104 (90.4)	53 (89.8)	
ALT, IU/L			0.769			0.996
≥2-fold normal upper limit	50 (22.9)	13 (22.0)		26 (22.6)	13 (22.0)	
0–2-fold normal upper limit	39 (17.9)	13 (22.0)		25 (21.7)	13 (22.0)	
Normal	129 (59.2)	33 (55.9)		64 (55.7)	33 (55.9)	
AST, IU/L			0.923			0.828
≥2-fold normal upper limit	59 (27.1)	16 (27.1)		35 (30.4)	16 (27.1)	
0–2-fold normal upper limit	57 (26.1)	14 (23.7)		29 (25.2)	14 (23.7)	
Normal	102 (46.8)	29 (49.2)		51 (44.3)	29 (49.2)	
MELD score			0.949			0.750
Median	9.0	9.0		9.0	9.0	
Interquartile range	6–13	5–14		6–14	5–14	
Infection			0.003			0.022
Present	40 (18.3)	22 (37.3)		23 (20.0)	22 (37.3)	
Absent	178 (81.7)	37 (62.7)		92 (80.0)	37 (62.7)	

*Data are presented as the number with percentage or median with interquartile range. ALT, alanine transaminase; AST, aspartate aminotransferase; BMI, body mass index; DM, diabetes mellitus; HDLC, high-density lipoprotein cholesterol; MELD, model for end-stage liver disease.*

**TABLE 4:** Clinical characteristics stratified by HDLC in patients with DM.

	Before propensity score matching			After propensity score matching		
	Low HDLC group (*n* = 59)	Normal HDLC group (*n* = 41)	*p*-value	Low HDLC group (*n* = 40)	Normal HDLC group (*n* = 31)	*p*-value
Sex			0.657			1.000
Male	41 (69.5)	31 (75.6)		28 (70.0)	22 (71.0)	
Female	18 (30.5)	10 (24.4)		13 (30.0)	9 (29.0)	
Age, years			0.068			0.600
≥60	21 (35.6)	23 (56.1)		17 (42.5)	16 (51.6)	
<60	38 (64.4)	18 (43.9)		23 (57.5)	15 (48.4)	
BMI, kg/m^2^			0.043			0.398
≥24.0	32 (54.2)	13 (31.7)		18 (45.0)	10 (32.3)	
<24.0	27 (45.8)	28 (68.3)		22 (55.0)	21 (67.7)	
Extra-hepatic disease			1.000			0.991
Present	19 (32.2)	14 (34.1)		13 (32.5)	11 (35.5)	
Absent	40 (67.8)	27 (65.9)		27 (67.5)	20 (64.5)	
Hepatocellular carcinoma			0.068			0.497
Present	17 (28.8)	20 (50.0)		15 (37.5)	15 (48.4)	
Absent	42 (71.2)	20 (50.0)		25 (62.5)	16 (51.2)	
Triglycerides, mmol/L			0.466*			1.000*
High	6 (10.2)	2 (4.9)		2 (5.0)	1 (3.2)	
Normal	53 (89.8)	39 (95.1)		38 (95.0)	30 (96.8)	
ALT, IU/L			0.055			0.996
≥2-fold normal upper limit	13 (22.0)	3 (9.8)		4 (10.00)	3 (9.7)	
0–2-fold normal upper limit	13 (22.0)	6 (17.1)		8 (20.0)	6 (19.4)	
Normal	33 (55.9)	32 (73.2)		28 (7)	22 (71.0)	
AST, IU/L			0.038			0.972
≥2-fold normal upper limit	16 (27.1)	4 (9.8)		5 (12.5)	4 (12.9)	
0–2-fold normal upper limit	14 (23.7)	7 (17.1)		10 (25.0)	7 (22.6)	
Normal	29 (49.2)	30 (73.2)		25 (62.5)	20 (64.5)	
MELD score			0.005			0.233
Median	9.0	6.0		8.0	6.0	
Interquartile range	5–14	4–9		5–12	4–10	
Infection			0.002			0.018
Present	22 (18.3)	3 (7.3)		13 (32.5)	2 (6.5)	
Absent	37 (81.1)	38 (92.7)		27 (67.5)	29 (93.5)	

Data are presented as the number with percentage or median with interquartile range. ALT, alanine transaminase; AST, aspartate aminotransferase; BMI, body mass index; DM, diabetes mellitus; HDLC, high-density lipoprotein cholesterol; MELD, model for end-stage liver disease. *Represents Fisher’s exact test.

Subsequently, types of BIs were analyzed in patients with diabetes and/or low HDLC (Supplementary Table 5). Intriguingly, there was no remarkable difference of types of BIs in patients with or without diabetes. While in patients with a low level of HDLC, respiratory and digestive system infections were more frequent (*p* = 0.025 and *p* = 0.006). Compared with those with low HDLC alone, patients with coexisting diabetes and low HDLC were found to have an increased risk of abdomen infections both before and after PSM (*p* = 0.022 and *p* = 0.044). Nonetheless, the types of BIs were comparable in patients with diabetes with or without the low level of HDLC.

## Discussion

Accumulative studies indicate that BIs are common in patients with cirrhosis and account for all-cause mortality (15). Not only the regenerative capacity of hepatocytes is impaired in cirrhotic livers for various reasons, but also portal hypertension and intestinal microbial translocation contribute to additional susceptibility for BIs (15, 16). Albeit the MELD scores are considered as the optimal tools for predicting mortality in patients with end-stage liver disease, the assessment of metabolic status is ignored. To date, most studies merely focus on the general epidemiology of pathogenic microorganisms in patients with cirrhosis (16–18). The risk imposed by abnormal metabolism has not yet been well-documented (8, 13).

In this study, metabolic risk factors of BIs, namely diabetes and low level of HDLC, were identified. Compared with individuals with diabetes or a low level of HDLC alone, patients with coexisting diabetes and a low level of HDLC showed higher risks of BIs. In line with previous reports (5, 8, 19), our study uncovered the nearly twofold higher risk of BIs in patients with diabetes. For patients with a low level of HDLC, the presence of diabetes further enhanced the risk of BIs, especially abdomen infections. However, the mechanisms underlying the synergy of diabetes and cirrhosis on BIs are unclear. Potentially, hyperglycemia disturbs the hemostasis of the immune system and provides a favorable microenvironment for bacterial growth (20). DM and cirrhosis are closely interrelated. Patients with cirrhosis should be screened for DM given its high prevalence in this population, especially in those with dyslipidemia. Continuous glucose monitoring is recommended for evaluating glucose control since the levels of glycated hemoglobin is not accurate in the presence of cirrhosis.

Of note, the increased incidence of BIs in patients with a low level of HDLC in this study is worthy of attention. In line with previous studies showing that patients with a low level of HDLC displayed a higher probability of BIs (21), our study revealed that patients with a low level of HDLC conferred a twofold higher risk of BIs than those with the normal level of HDLC. By scavenging cholesterol levels, HDLC attenuates endotoxins- or macrophages-mediated inflammatory response (22). Therefore, exogenous supplementation of HDLC could be a promising therapeutic target, and the definite effect of preventing BIs in patients with cirrhosis remains to be further investigated. Although the benefit of tight glycemic control in cirrhosis has never been demonstrated, the detrimental effects of a low level of HDLC had been shown in patients with chronic liver failure (13). The levels of HDLC can be easily measured in clinical laboratories and should be strongly recommended during the follow-up of patients with cirrhosis.

Intriguingly, no relationship between TG and BIs was observed in our cohort. Triglyceride, a component of blood lipids related to cardiovascular disease and diabetes, is influenced by lifestyle, diet, age, sex, and fluctuated widely and individually (23). We speculated that, as the number of patients with high TG is limited, the impact of TG on BIs could not be fully elucidated due to the inadequate power of the statistical test. It should be noted that the hepatic triglyceride content may be a better indicator of the severity of the fatty liver disease. Given its high prevalence in patients with chronic HBV infection, fatty liver disease is recognized to coexist with other metabolic dysfunctions. Although magnetic resonance imaging proton density fat fraction (MRI-PDFF) has been recommended to quantify hepatic fat content and to assess treatment response (24), the quantification of fat deposition may be influenced by liver nodularity in patients with cirrhosis. It is necessary to explore the quantification of fat content in cirrhosis through matching liver pathology histology with MRI.

Additionally, obesity implies a chronic metabolic disturbance with excessive body fat accumulation, which is one of the most prevalent manifestations of metabolic syndrome (25). Some studies have shown that obesity and metabolic disorders are accompanied by chronic low-grade inflammation. Plenty of clinical trials has uncovered the closed association between overweight and infections (26–28). However, there was no association between BMI and BI in our cohort. It is speculated that patients with cirrhosis may have a combination of ascites and edema, and therefore, the nutritional status could not be accurately reflected by BMI. Therefore, BMI is controversial to precisely evaluate the possibility of BIs in such patients.

At present, the Child-Turcotte-Pugh (CTP) score and the model for end-stage liver disease (MELD) scores are considered as the optimal tools for predicting mortality in patients with cirrhosis. Despite both scores simply obtained from clinical and laboratory data, one of the limitations of these scores is the lack of assessment of metabolic status. Further studies are warranted to confirm whether the incorporation of specific metabolic factors would improve the clinical utility of prognostic scores in predicting the outcome of patients with cirrhosis. In addition, glucose and lipid assessment should be started early and may be mandatory in the disease course in any patients with cirrhosis.

It is important to emphasize that patients with liver cancer in this cohort accounted for a higher proportion than expected. The authors feel that the management of the major complications of cirrhosis should not only be the job of internists. Patients with hepatectomy for HCC also require optimal management of cirrhotic complications in addition to assessing tumor recurrence. Blood glucose and HDLC level should be under careful supervision to prevent further decompensated cirrhosis caused by BIs. Therefore, the inclusion of patients with stable HCC with HBV-related cirrhosis fits with real-world conditions and facilitates comprehensive management of patients with cirrhosis. This research would help to improve current care pathways and the overall outcomes in these patients.

Some limitations of this study should be acknowledged. As a single-center retrospective study over a 10-year period, selected bias could not be completely avoided, although PSM enables the accurate assessment *via* minimizing the impacts of confounding factors. This study excluded patients with missing data, which also introduce selection bias. Second, too much data on mortality were missing. The differences in mortality between groups were not analyzed. Third, patients enrolled in this study were HBV-related cirrhosis; whether these findings can be replicated in patients with other etiologies of cirrhosis remain to be further investigated. Finally, the relationships between metabolism factors and length of stay and the long-term prognosis of these patients should be further investigated.

In conclusion, our study found that diabetes and low level of HDLC were the risk factors of BIs in patients with HBV-related cirrhosis. Moreover, the coexistence of diabetes and a low level of HDLC further enhanced the risk of BIs. Screening for glucose and lipid metabolism should be initiated during hospitalization in these patients.

## Data Availability Statement

The original contributions presented in the study are included in the article/Supplementary Material, further inquiries can be directed to the corresponding author/s.

## Ethics Statement

The Ethics Code: No. 20200522-28, approved by the Ethics Review and Scientific Investigation Board of Sir Run Run Shaw Hospital, School of Medicine, Zhejiang University (Hangzhou, Zhejiang, China). Written informed consent for participation was not required for this study in accordance with the national legislation and the institutional requirements.

## Author Contributions

FL and QY conceived and designed the manuscript. YT and HY collected and analyzed the data. QY and YT wrote the manuscript. BP and FL revised and checked the manuscript. All authors contributed to the article and approved the submitted version.

## Conflict of Interest

The authors declare that the research was conducted in the absence of any commercial or financial relationships that could be construed as a potential conflict of interest.

## Publisher’s Note

All claims expressed in this article are solely those of the authors and do not necessarily represent those of their affiliated organizations, or those of the publisher, the editors and the reviewers. Any product that may be evaluated in this article, or claim that may be made by its manufacturer, is not guaranteed or endorsed by the publisher.

## Footnotes

1
www.R-project.org

## Abbreviations

ACLF: acute-on-chronic liver failure
BIs: bacterial infections
BMI: body mass index
DM: diabetes mellitus
HBV: hepatitis B virus
HDLC: high-density lipoprotein cholesterol
MELD: model for end-stage liver disease
NAFLD: non-alcoholic fatty liver disease
PSM: propensity score matching
TG: triglyceride.
