# Supplementary material for: Influence of Metabolic Risk Factors on the Risk of Bacterial Infections in Hepatitis B-Related Cirrhosis: A 10-Year Cohort Study
Source: Front Med (Lausanne). 2022 Apr 14;9:847091. doi: 10.3389/fmed.2022.847091 (PMC9046983; doi:10.3389/fmed.2022.847091)
Supplement: Supplementary file 1 [file Table_1.DOCX]

**Supplementary Table 1 Definition for bacterial infections**

| Bacterial infection was diagnosed according to conventional criteria ^[13]^ | |
| --- | --- |
| Bacteremia | Bacteremia was defined as the growth of an uncommon skin contaminant in more than one blood culture and of a common skin contaminant (e.g., diphtheroids, Bacillus species, coagulase-negative staphylococci, Propionibacterium species or micrococci) in more than two blood cultures drawn from separate sites, with signs of infection |
| Pneumonia | New radiological pulmonary infiltration with the presence of: (i) dyspnea, cough, purulent sputum, pleuritic chest pain or signs of consolidation (ii) positive findings on auscultation (rales or crepitation) or at least one sign of infection: core body temperature >38℃ or <36℃, orleukocyte count>10,000/mm^3^ or <4,000/mm^3^ in the absence of antibiotics |
| Bacterial enterocolitis | Diarrhea with an increase in fecal white blood cell counts ≥15/high-power field or a positive stool culture for Salmonella, Shigella, Yersinia, Campylobacter, or pathogenic E. coli |
| Urinary tract infection | Urine white blood cell >15/high-power field with either positive urine culture or urinary irritation |
| Spontaneous bacterial peritonitis (SBP) | Polymorphonuclear cells in ascitic fluid > 250/uL |
| Others | Including skin infection, intra-abdominal infections and unproven infection |

| Supplement Table 2 Characteristics of all patients | | | |
| --- | --- | --- | --- |
|  | Non-infection group (n=390) | Infection group (n=81) | *p*-value |
| Sex |  |  | 0.203 |
| Male | 296 (75.9) | 56 (69.1) |  |
| Female | 94 (24.1) | 25 (30.9) |  |
| Age, years |  |  | 0.027 |
| ≥ 60 | 152 (39.0) | 21 (25.9) |  |
| < 60 | 238 (S61.0) | 60 (74.1) |  |
| BMI, kg/m^2^ |  |  | 0.902 |
| ≥ 24.0 | 156 (40.0) | 33 (40.7) |  |
| < 24.0 | 234 (60.0) | 48 (59.3) |  |
| Diabetes |  |  | 0.020 |
| Present | 75 (19.2) | 25 (30.9) |  |
| Absent | 315 (80.8) | 56 (69.1) |  |
| Extra-hepatic disease |  |  | 0.666 |
| Present | 97 (24.9) | 22 (27.2) |  |
| Absent | 293 (75.1) | 59 (72.8) |  |
| Hepatocellular carcinoma |  |  | 0.005 |
| Present | 167 (42.8) | 21 (25.9) |  |
| Absent | 223 (57.2) | 60 (74.1) |  |
| HDLC, mmol/L |  |  | <0.001 |
| Normal | 175 (44.9) | 19 (23.5) |  |
| Low | 215 (55.1) | 62 (76.5) |  |
| Triglycerides, mmol/L |  |  | 0.118 |
| High | 35 (9.0) | 6 (7.4) |  |
| Normal | 355 (91.0) | 75 (92.6) |  |
| ALT, IU/L |  |  | 0.008 |
| ≥ 2 fold normal upper limit | 55 (14.1) | 22 (27.2) |  |
| 0-2 fold normal upper limit | 78 (20.0) | 18 (22.2) |  |
| Normal | 257 (65.9) | 41 (50.6) |  |
| AST, IU/L |  |  | <0.001 |
| ≥ 2 fold normal upper limit | 59 (15.1) | 29 (35.8) |  |
| 0-2 fold normal upper limit | 112 (28.7) | 21 (25.9) |  |
| Normal | 219 (56.2) | 31 (38.3) |  |
| MELD score |  |  | <0.001 |
| Median | 8.0 | 10.0 |  |
| Interquartile range | 5-11 | 7-15 |  |
| Data are presented as number with percentage or median with interquartile range. ALT: Alanine transaminase; AST: Aspartate aminotransferase; BMI: Body mass index; DM: diabetes mellitus; HDLC: High density lipoprotein cholesterol; MELD: Model for end stage liver disease. | | | |

| Supplement Table 3 Clinical characteristics stratified by BMI | | | | | | |
| --- | --- | --- | --- | --- | --- | --- |
|  | Before Propensity Score Matching | | | After Propensity Score Matching | | |
|  | Lean group  (n=282) | Overweight group  (n=189) | *p*-value | Lean group  (n=267) | Overweight group  (n=171) | *p*-value |
| Sex |  |  | 0.871 |  |  | 0.690 |
| Male | 212 (75.2) | 140 (74.1) |  | 201 (75.3) | 125 (73.1) |  |
| Female | 71 (24.8) | 49 (25.9) |  | 66 (24.7) | 46 (26.9) |  |
| Age, years |  |  | 0.167 |  |  | 0.591 |
| ≥ 60 | 96 (34.0) | 77 (40.7) |  | 95 (35.6) | 66 (38.6) |  |
| < 60 | 186 (66.0) | 122 (59.3) |  | 172 (64.4) | 105 (61.4) |  |
| Diabetes |  |  | 0.315 |  |  | 0.295 |
| Present | 55 (19.5) | 45 (23.8) |  | 53 (19.9) | 42 (24.6) |  |
| Absent | 227 (80.5) | 144 (76.2) |  | 214 (80.1) | 129 (75.4) |  |
| Extra-hepatic disease |  |  | 0.001 |  |  | 0.188 |
| Present | 56 (19.9) | 63 (33.3) |  | 56 (21.0) | 46 (26.9) |  |
| Absent | 226 (80.1) | 126 (66.7) |  | 211 (79.0) | 125 (73.1) |  |
| Hepatocellular carcinoma |  |  | 0.254 |  |  | 0.250 |
| Present | 119 (42.2) | 69 (36.5) |  | 108 (40.4) | 59 (34.5) |  |
| Absent | 163 (57.8) | 120 (63.5) |  | 159 (59.6) | 112 (65.5) |  |
| HDLC, mmol/L |  |  | 0.006 |  |  | 0.089 |
| Normal | 131 (46.5) | 63 (33.3) |  | 120 (44.9) | 62 (36.3) |  |
| Low | 151 (53.5) | 126 (66.7) |  | 147 (55.1) | 109 (63.7) |  |
| Triglycerides, mmol/L |  |  | 0.495 |  |  | 0.710 |
| High | 22 (7.8) | 19 (10.1) |  | 21 (7.9) | 16 (9.4) |  |
| Normal | 260 (92.2) | 170 (89.9) |  | 246 (92.1) | 155 (90.6) |  |
| ALT, IU/L |  |  | 0.157 |  |  | 0.302 |
| ≥ 2 fold normal upper limit | 39 (13.8) | 38 (20.1) |  | 39 (14.6) | 34 (19.9) |  |
| 0-2 fold normal upper limit | 62 (22.0) | 34 (18.0) |  | 54 (20.2) | 29 (17.0) |  |
| Normal | 181 (64.2) | 117 (61.9) |  | 174 (65.2) | 108 (63.2) |  |
| AST, IU/L |  |  | 0.856 |  |  | 0.782 |
| ≥ 2 fold normal upper limit | 53 (18.8) | 35 (18.5) |  | 49 (18.4) | 35 (20.5) |  |
| 0-2 fold normal upper limit | 77 (27.3) | 56 (29.6) |  | 72 (27.0) | 48 (28.1) |  |
| Normal | 152 (53.9) | 98 (51.9) |  | 146 (54.7) | 88 (51.5) |  |
| MELD score |  |  | 0.017 |  |  | 0.253 |
| Median | 8.0 | 9.0 |  | 8.0 | 9.0 |  |
| Interquartile range | 5-11 | 5-13 |  | 5-12 | 5-13 |  |
| Infection |  |  | 1.000 |  |  | 0.532 |
| Present | 48 (17.0) | 33 (17.5) |  | 44 (16.5) | 29 (17.0) |  |
| Absent | 234 (83.0) | 156 (82.5) |  | 223 (83.5) | 142 (83.0) |  |
| Data are presented as number with percentage or median with interquartile range. Lean group: BMI<24.0 kg/m^2^, Overweight group: BMI≥24.0 kg/m^2^; ALT: Alanine transaminase; AST: Aspartate aminotransferase; BMI: Body mass index; DM: diabetes mellitus; HDLC: High density lipoprotein cholesterol; MELD: Model for end stage liver disease. | | | | | | |

| Supplement Table 4 Clinical characteristics stratified by TG | | | | | | |
| --- | --- | --- | --- | --- | --- | --- |
|  | Before Propensity Score Matching | | | After Propensity Score Matching | | |
|  | Normal TG group  (n=430) | High TG group  (n=41) | *p*-value | Normal TG group  (n=73) | High TG group  (n=38) | *p*-value |
| Sex |  |  | 1.000 |  |  | 1.000 |
| Male | 321 (74.7) | 31 (75.6) |  | 57 (78.1) | 29 (76.3) |  |
| Female | 109 (25.3) | 10 (24.4) |  | 16 (21.9) | 9 (23.7) |  |
| Age, years |  |  | 0.597 |  |  | 0.295 |
| ≥ 60 | 160 (37.2) | 13 (31.7) |  | 32 (43.8) | 12 (31.6) |  |
| < 60 | 270 (62.8) | 28 (68.3) |  | 41 (56.2) | 26 (68.4) |  |
| BMI, kg/m^2^ |  |  | 0.495 |  |  | 0.887 |
| ≥ 24.0 | 170 (39.5) | 19 (46.3) |  | 34 (46.6) | 19 (50.0) |  |
| < 24.0 | 260 (60.5) | 22 (53.7) |  | 39 (53.4) | 19 (50.0) |  |
| Diabetes |  |  | 0.935 |  |  | 0.734 |
| Present | 92 (21.4) | 8 (19.5) |  | 12 (16.4) | 8 (21.1) |  |
| Absent | 338 (78.6) | 33 (80.5) |  | 61 (83.6) | 30 (78.9) |  |
| Extra-hepatic disease |  |  | 0.421 |  |  | 0.836 |
| Present | 106 (38.6) | 13 (31.7) |  | 24 (32.9) | 11 (28.9) |  |
| Absent | 324 (61.4) | 28 (68.3) |  | 49 (67.1) | 27 (71.1) |  |
| Hepatocellular carcinoma |  |  | 0.087 |  |  | 0.676 |
| Present | 166 (38.6) | 22 (53.7) |  | 32 (43.8) | 19 (50.0) |  |
| Absent | 264 (61.4) | 19 (46.3) |  | 41 (56.2) | 19 (50.0) |  |
| HDLC, mmol/L |  |  | 0.034 |  |  | 0.905 |
| Normal | 184 (42.8) | 10 (24.4) |  | 17 (23.3) | 10 (26.3) |  |
| Low | 246 (57.2) | 31 (75.6) |  | 56 (76.7) | 28 (73.7) |  |
| ALT, IU/L |  |  | 0.014 |  |  | 0.876 |
| ≥ 2 fold normal upper limit | 64 (14.9) | 13 (31.7) |  | 26 (35.6) | 12 (31.6) |  |
| 0-2 fold normal upper limit | 87 (20.2) | 9 (22.0) |  | 16 (21.9) | 8 (21.1) |  |
| Normal | 279 (64.9) | 19 (46.3) |  | 31 (42.5) | 18 (47.4) |  |
| AST, IU/L |  |  | 0.008 |  |  | 0.310 |
| ≥ 2 fold normal upper limit | 73 (17.0) | 15 (36.6) |  | 31 (42.5) | 13 (34.2) |  |
| 0-2 fold normal upper limit | 125 (29.1) | 8 (19.5) |  | 20 (27.4) | 8 (21.1) |  |
| Normal | 232 (54.0) | 18 (43.9) |  | 22 (30.1) | 17 (44.7) |  |
| MELD score |  |  | 0.022 |  |  | 0.267 |
| Median | 9.0 | 7.0 |  | 8.0 | 7.0 |  |
| Interquartile range | 5-12 | 4-10 |  | 6-11 | 4-10 |  |
| Infection |  |  | 0.811 |  |  | 0.404 |
| Present | 75 (17.4) | 6 (14.6) |  | 18 (24.7) | 6 (15.8) |  |
| Absent | 355 (82.6) | 35 (85.4) |  | 55 (75.3) | 32 (84.2) |  |
| Data are presented as number with percentage or median with interquartile range. ALT: Alanine transaminase; AST: Aspartate aminotransferase; BMI: Body mass index; HDLC: High density lipoprotein cholesterol; MELD: Model for end stage liver disease; TG: Triglycerides. | | | | | | |

| Supplement Table 5 Type of infection | | | | | | |
| --- | --- | --- | --- | --- | --- | --- |
|  | Before Propensity Score Matching | | | After Propensity Score Matching | | |
| In all patients | Non-diabetes group  (n=371) | Diabetes group  (n=100) | *p*-value | Non-diabetes group  (n=188) | Diabetes group  (n=95) | *p*-value |
| Bacteremia | 4 (1.1) | 1 (1.0) | 1.000* | 2 (1.1) | 1 (1.1) | 1.000* |
| Pneumonia | 24 (6.5) | 9 (9.0) | 0.510 | 8 (4.3) | 8 (8.4) | 0.246 |
| Bacterial enterocolitis | 9 (2.4) | 5 (5.0) | 0.188* | 6 (3.2) | 5 (5.3) | 0.517 |
| Urinary system | 6 (1.6) | 2 (2.0) | 0.680* | 3 (1.6) | 2 (2.1) | 1.000* |
| SPB | 22 (5.9) | 11 (11.0) | 0.123 | 12 (6.4) | 10 (10.5) | 0.320 |
| Others | 7 (1.9) | 4 (4.0) | 0.257* | 2 (1.1) | 4 (4.2) | 0.100* |
| In all patients | Low HDLC group  (n=277) | Normal HDLC group  (n=194) | *p*-value | Low HDLC group  (n=214) | Normal HDLC group  (n=164) | *p*-value |
| Bacteremia | 3 (1.1) | 2 (1.0) | 1.000* | 0 (0) | 2 (1.2) | 0.188* |
| Pneumonia | 28 (10.1) | 5 (2.6) | 0.003 | 18 (8.4) | 4 (2.4) | 0.025 |
| Bacterial enterocolitis | 14 (5.1) | 0 (0) | 0.004 | 9 (4.2) | 0 (0) | 0.006* |
| Urinary system | 4 (1.4) | 4 (2.1) | 0.722* | 2 (0.9) | 3 (1.8) | 0.656* |
| SBP | 24 (8.7) | 9 (4.6) | 0.133 | 14 (6.5) | 8 (4.9) | 0.643 |
| Others | 8 (2.9) | 3 (1.5) | 0.537* | 3 (1.4) | 2 (1.2) | 1.000* |
| In patients with low HDLC | Non-diabetes group  (n=218) | Diabetes group  (n=59) | *p*-value | Non-diabetes group  (n=115) | Diabetes group  (n=59) | *p*-value |
| Bacteremia | 3 (1.4) | 0 (0) | 1.000* | 3 (2.6) | 0 (0) | 0.552* |
| Pneumonia | 20 (9.2) | 8 (13.6) | 0.455 | 10 (8.7) | 8 (13.6) | 0.463 |
| Bacterial enterocolitis | 9 (4.1) | 5 (8.5) | 0.186* | 8 (7.0) | 5 (8.5) | 0.765* |
| Urinary system | 2 (0.9) | 2 (3.4) | 0.200* | 1 (0.9) | 2 (3.4) | 0.266* |
| SBP | 14 (6.4) | 10 (16.9) | 0.022 | 7 (6.1) | 10 (16.9) | 0.044 |
| Others | 5 (2.3) | 3 (5.1) | 0.375* | 3 (2.6) | 3 (5.1) | 0.409* |
| In patients with diabetes | Low HDLC group  (n=59) | Normal HDLC group  (n=41) | *p*-value | Low HDLC group  (n=40) | Normal HDLC group  (n=31) | *p*-value |
| Bacteremia | 0 (0) | 1 (2.4) | 0.410* | 0 (0) | 0 (0) | NA |
| Pneumonia | 8 (13.6) | 1 (2.4) | 0.078* | 6 (15.0) | 1 (3.2) | 0.128* |
| Bacterial enterocolitis | 5 (8.5) | 0 (0) | 0.076* | 3 (7.5) | 0 (0) | 0.252* |
| Urinary system | 2 (3.4) | 0 (0) | 0.511* | 1 (2.5) | 0 (0) | 1.000* |
| SBP | 10 (16.9) | 1 (2.4) | 0.025* | 5 (2.5) | 1 (3.2) | 0.222* |
| Others | 3 (5.1) | 1 (2.4) | 0.642* | 2 (5.0) | 0 (0) | 0.501* |
| Data are presented as number with percentage. HDLC: High density lipoprotein cholesterol. SBP: Spontaneous bacterial peritonitis. Others: including skin infection, intra-abdominal infections and unproven infections. NA: Not Applicable. * represent the Fisher exact test. | | | | | | |
